# Supplementary material for: Prolonging somatic cell proliferation through constitutive hox gene expression in C. elegans
Source: Nat Commun. 2023 Oct 27;14:6850. doi: 10.1038/s41467-023-42644-1 (PMC10611754; doi:10.1038/s41467-023-42644-1)
Supplement: Supplementary file 1 — Supplementary Information [file 41467_2023_42644_MOESM1_ESM.pdf]

Supplementary information

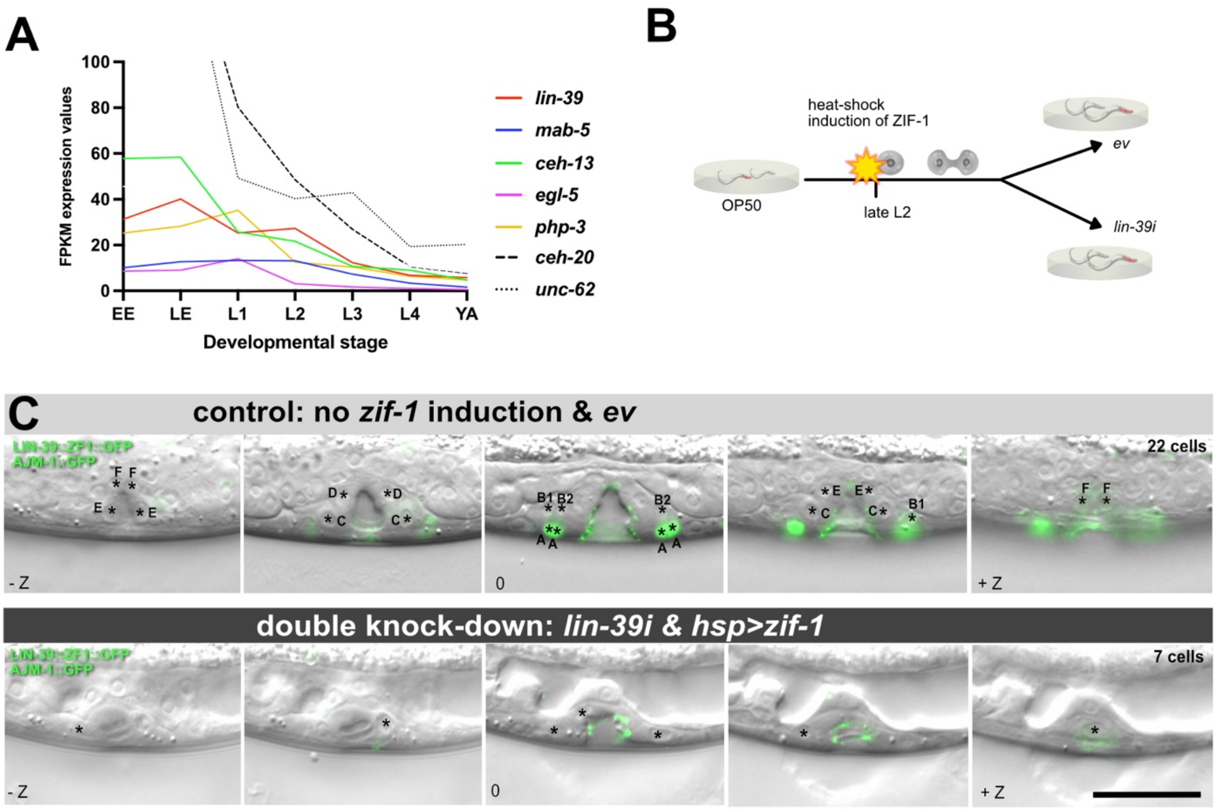

Figure S1 related to Figure 1.

(A) Developmental mRNA expression profiles of the six *hox* genes and the co-factors *unc-62* and *ceh-20* based on data from the modEncode project [15]. FPKM = Fragments Per Kilobase of transcript per Million mapped reads. (**B**) Schematic representation of the procedure for the conditional *lin-39* knock-down experiments. *zh120(lin-39::zf1::gfp); zhEx616[hsp-16>zif-1]* larvae were heat-shocked for 50 minutes at 31°C at the late L2 stage (24h post L1 starvation arrest) and subsequently grown at 20°C on *lin-39* dsRNA-producing (*lin-39i*) or empty vector (ev) control bacteria, as indicated in **Fig. 1C**. (**C**) Individual z-sections of the control and double knock-down L4 larvae shown in **Fig. 1B**. DIC images overlaid with the AJM-1::GFP (*swIs79*) and LIN-39::GFP (*zh120*) signals (green) are shown. Vulval cell nuclei are marked with black asterisks and the numbers of vulval cells per animal are indicated at the top right of the last section. Scale bar is 20 µm.

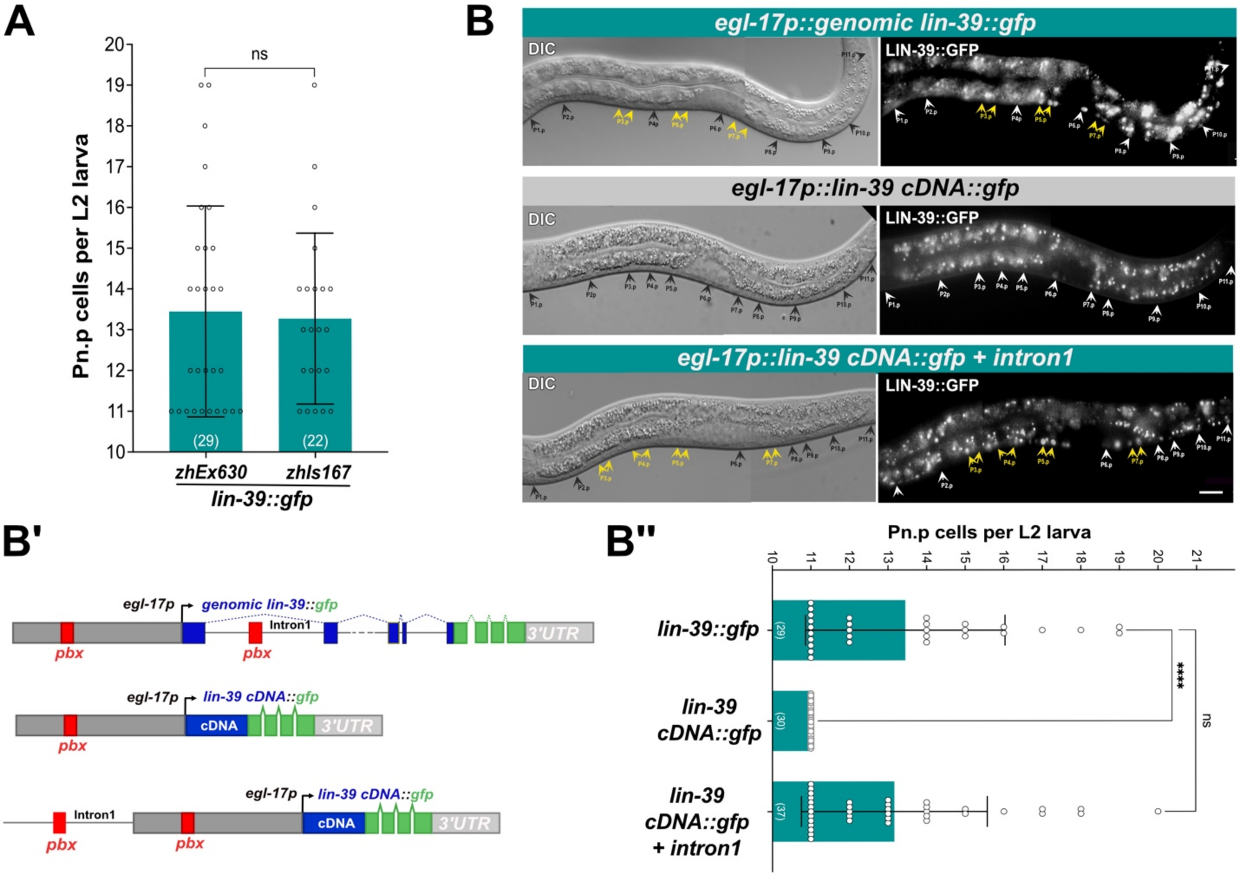

Figure S2 related to Figure 2A.

(A) Comparison of the early Pn.p cell duplication phenotype in extra-chromosomal *zhEx630[egl-17p>lin-39::gfp]* versus integrated *zhIs167[egl-17p>lin-39::gfp]* L2 larvae. **(B)** The first intron of *lin-39* is necessary for early Pn.p cell duplication. Undivided Pn.p cell nuclei are marked with black (DIC) and white arrows (GFP), yellow arrows indicate duplicated Pn.p cells. The scale bar is 20 µm. **(B')** Schematic drawings of the different transgenes used. (**B''**) Quantification of Pn.p cell numbers in the different transgenic lines. Three independent *[egl-17p>lin-39::gfp]* (*zhEx630.1-630.3*), two *[egl-17p>lin-39cDNA::gfp]* (*zhEx641.1&642.2*) and three *[egl-17p>lin-39cDNA::gfp +intron1]* lines (*zhEx642.1-642.3*) were analyzed. Bars in (**A**) and (**B''**) show the average numbers of Pn.p cells, error bars indicate the standard deviation and dots the individual values. Numbers of animals scored are indicated in brackets. Statistical significance was determined with a Kruskal-Wallis non-parametric test and is indicated with ns for $p>0.05$ and **** for $p = 5.1e-5$.

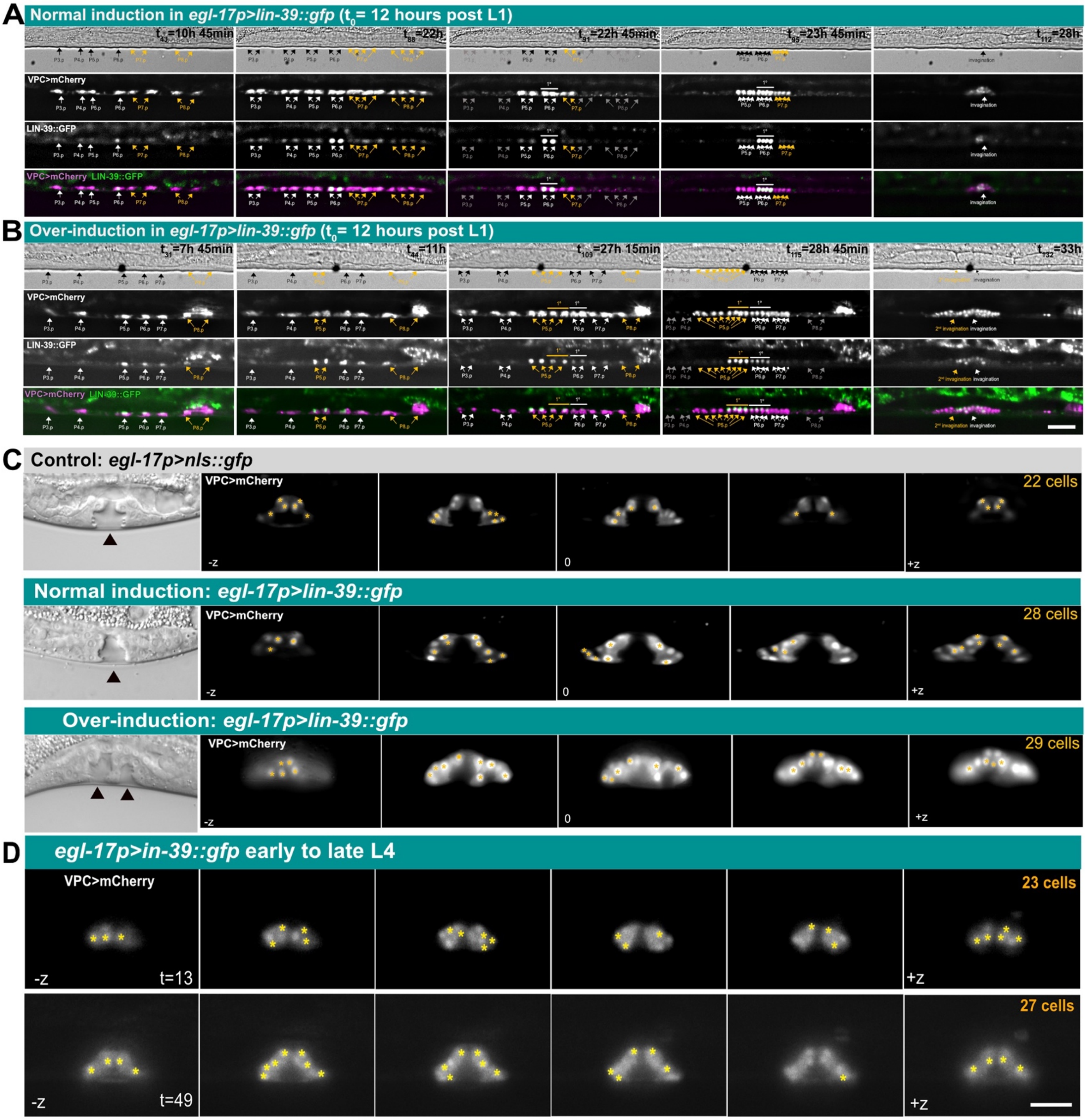

E

Animal no.:	1	**2**	3	4	5	**6**	7	8	9	10	11	12	**13**	14	15	16	**17**	18	19	20	**21**
early L4 no. vulval cells	22	22	22	22	24	24	22	22	22	24	28	23	22	27	22	22	23	22	22	22	22
late L4 no. vulval cells	22	26	26	22	24	28	22	22	22	24	28	23	25	27	22	22	27	22	22	22	25

Figure S3 related to Figure 2B and 2C.

(**A**) Time-lapse imaging sequence from the mid-L2 until the L4.1 stage of a *zhIs167[egl-17p>lin-39::gfp]* larva with early Pn.p cell duplications but normal vulval induction and (**B**) a *zhIs167* larva with early Pn.p cell duplications and over-induction leading to a second vulval invagination. Time-points are indicated relative to the start of the imaging experiment (t_0) 12 hours post L1 starvation arrest. Images were taken at 15-minute intervals. Non-duplicated Pn.p cells and their descendants are marked with white arrows and duplicated Pn.p cells and their descendants with yellow arrows. Cells adopting the 1° fate based on strong LIN-39::GFP expression at the Pn.pxx stage are indicated with white and yellow lines. See **Movies S2 & S3** for all time-points. (**C**) Individual z-sections of the larvae shown in **Fig. 2B**. Black arrowheads in the DIC images of mid-sagittal sections indicate the vulval invaginations. Vulval cell nuclei expressing the VPC>mCherry reporter are indicated once with yellow asterisks, and the total number of vulval cells counted across the z-stack is indicated at the top of the last z-section (+z). (**D**) Vulval cell counts in a *zhIs167[egl-17p>lin-39::gfp]* larva at the early L4.2 (top) and late L4.7 (bottom) stage . Individual z-sections of the vulval cells labelled with the VPC>mCherry reporter are shown. Cell nuclei are marked once with yellow asterisks, and the total numbers of vulval cells counted across the z-sections are indicated at the top of the last z-section (+z). (**E**) Table summarizing the results of the time-lapse analysis of twenty-one *zhIs167* animals between the early (L4.1-L4.2 substages) and late (L4.7 toL4.8 substages) L4 stage. Animals, in which the vulval cell numbers increased from early to late L4, are highlighted in bold. (**D)** Animal number **17** at early and late L4. Scale bars are 20 μm.

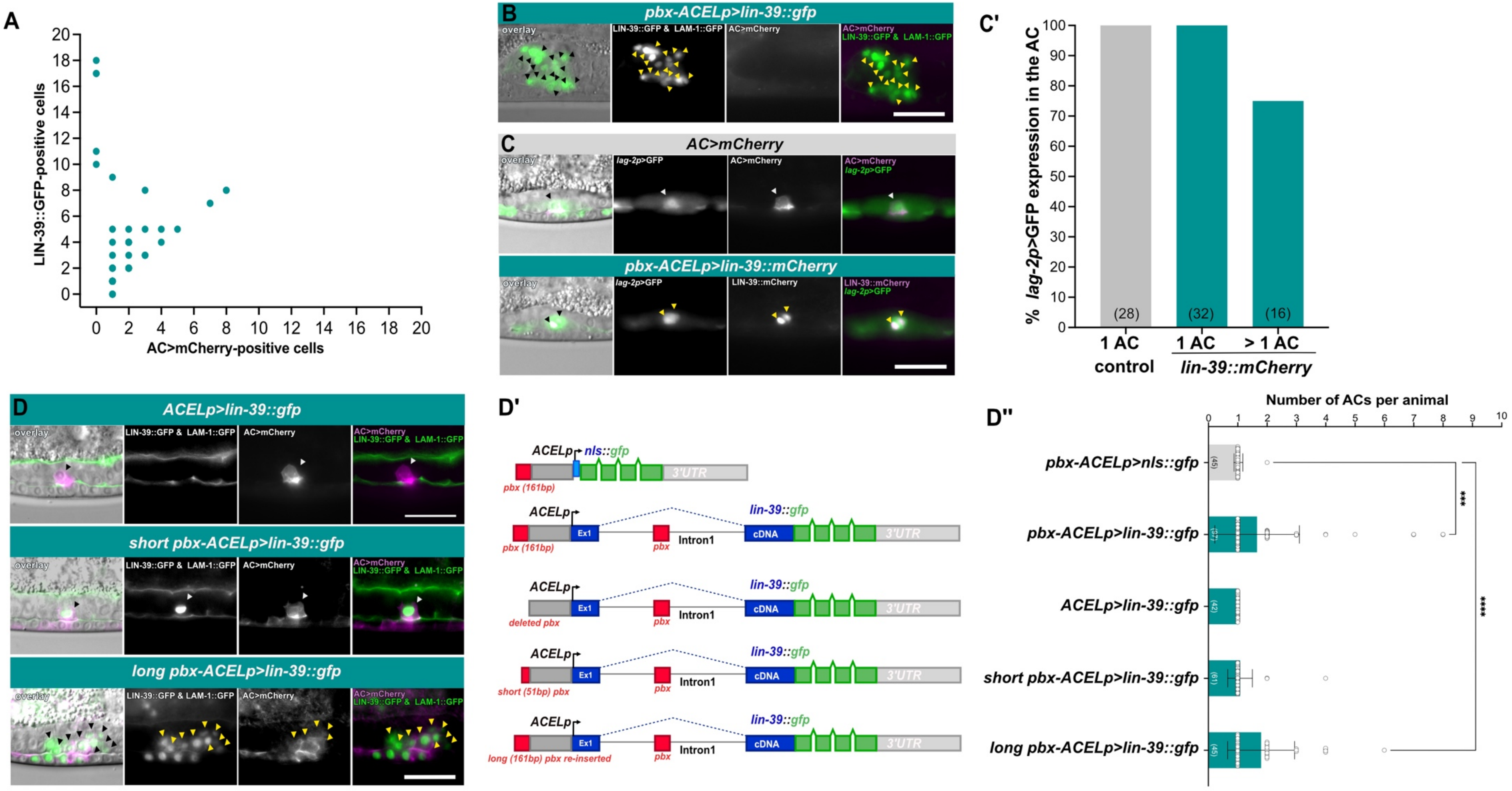
A
LIN-39::GFP-positive cells
AC>mCherry-positive cells
B
pbx-ACELp>lin-39::gfp
overlay
LIN-39::GFP & LAM-1::GFP
AC>mCherry
AC>mCherry LIN-39::GFP & LAM-1::GFP
C
AC>mCherry
lag-2p>GFP
AC>mCherry lag-2p>GFP
pbx-ACELp>lin-39::mCherry
LIN-39::mCherry
LIN-39::mCherry lag-2p>GFP
C'
% lag-2p>GFP expression in the AC
(28)
(32)
(16)
1 AC control
1 AC
> 1 AC
lin-39::mCherry
D
ACELp>lin-39::gfp
short pbx-ACELp>lin-39::gfp
long pbx-ACELp>lin-39::gfp
D'
ACELp
nls::gfp
3'UTR
pbx (161bp)
Ex1
pbx
Intron1
lin-39::gfp
cDNA
deleted pbx
short (51bp) pbx
long (161bp) pbx re-inserted
D''
Number of ACs per animal
pbx-ACELp>nls::gfp
pbx-ACELp>lin-39::gfp
ACELp>lin-39::gfp
short pbx-ACELp>lin-39::gfp
long pbx-ACELp>lin-39::gfp
***
****

Figure S4 related to Figure 4.

(**A**) Correlation between LIN-39::GFP (y-axis) and AC>mCherry (*qyIs23*) (x-axis) expression in *pbx-ACELp>lin-39::gfp* animals. Each dot indicates the numbers of LIN-39::GFP- and AC>mCherry-positive cells per L3 or L4 larva. Two independent *pbx-ACELp>lin-39::gfp* lines (*zhEx650.1* & *650.2*) were analyzed. (**B**) Loss of AC>mCherry (*qyIs23*) expression in a *zhEx650[pbx-ACELp>lin-39::gfp]* larva containing sixteen LIN-39::GFP-positive cells. (**C**) *lag-2p*>GFP (*qIs56*) expression is the ACs of an AC>mCherry (*qyIs23*) control (top) and a *zhEx689[pbx-ACELp>lin-39::mCherry]* L3 larva (bottom). (**C′**) Quantification of *lag-2p*>GFP expression in AC>mCherry control and *zhEx689[pbx-ACELp>lin-39::mCherry]* L3 and L4 larvae, divided into animals with a single and multiple LIN-39::mCherry-positive cells. Two independent *pbx-ACELp>lin-39::mCherry* lines (*zhEx689.1*& *689.2*) were analyzed. (**D**) AC proliferation in animals carrying the modified *pbx-ACELp>lin-39::gfp* transgenes illustrated in (**D′**). (**D′′**) Quantification of AC proliferation in the transgenic lines shown in (**D**, **D′**). Two independent lines were analyzed for each transgene: *ACELp>lin-39::gfp* (*zhEx664.1 & 664.2), short-pbx-ACELp>lin-39::gfp* (*zhEx665.1 & 665.2)* and *long-pbx-ACELp>lin-39::gfp* (*zhEx650.1& 650.2)*. Arrowheads in (**B**), (**C**) & (**D**) indicate AC nuclei. Black arrowheads in DIC images and white arrowheads in the fluorescent images indicate single and yellow arrowheads multiple ACs.) The bar plot (**D′′**) shows the average number of ACs in each population, error bars indicate the standard deviation and dots the individual values. Numbers of animals scored are indicated in brackets. Statistical significance was determined with a Kruskal-Wallis non-parametric test followed by Dunn's multiple comparison correction and is indicated with *** for $p = 0.0002$ and **** for $p = 3.7e-7$. The scale bars are 20 μm.

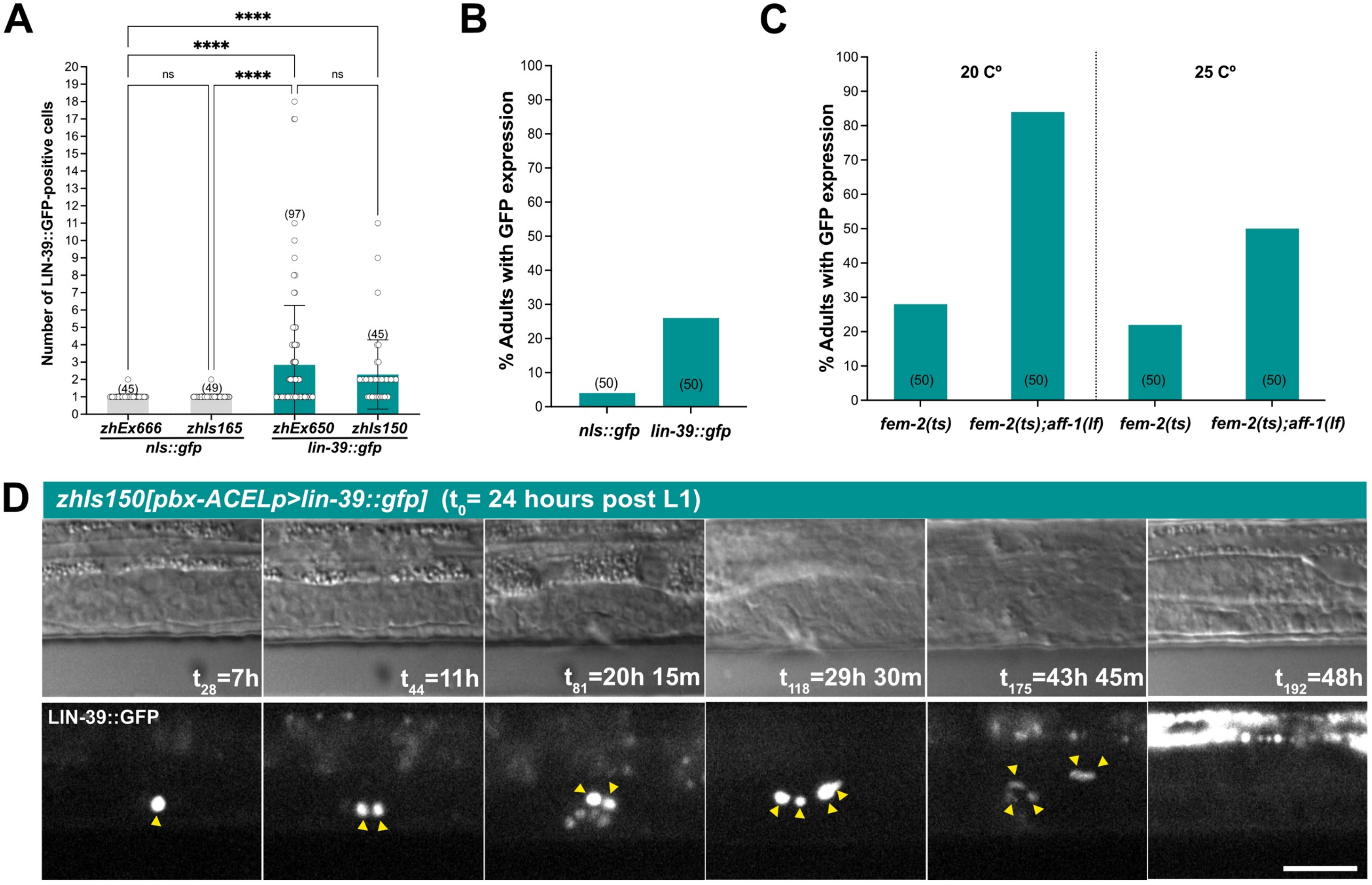
A
B
C
D
Number of LIN-39::GFP-positive cells
****
****
ns
****
ns
(45)
(49)
(97)
(45)
zhEx666
zhIs165
nls::gfp
zhEx650
zhIs150
lin-39::gfp
% Adults with GFP expression
(50)
nls::gfp
lin-39::gfp
20 C°
25 C°
fem-2(ts)
fem-2(ts);aff-1(lf)
zhIs150[pbx-ACELp>lin-39::gfp] (t0= 24 hours post L1)
t28=7h
t44=11h
t81=20h 15m
t118=29h 30m
t175=43h 45m
t192=48h
LIN-39::GFP

Figure S5 related to Figure 4.

(A) Comparison of AC proliferation in in L3 larvae containing integrated versus extra-chromosomal versions of the *pbx-ACELp>nls::gfp* and *pbx-ACELp>lin-39::gfp* transgenes. Two independent extra-chromosomal lines were analyzed for each transgene (*pbx-ACELp>nls::gfp*: *zhEx666.1 & 666.2, pbx-ACELp>lin-39::gfp*: *zhEx650.1 & 650.2).* (**B**) Frequency of NLS::GFP (*zhIs165*) and LIN-39::GFP (*zhIs150*) expression in one day-old adult animals. (**C**) Frequency of LIN-39::GFP expression in one day-old *zhIs150* adult animals carrying the *fem-2*(*b245ts*) and *aff-1*(*tm2114*) mutations and grown from the L1 stage on at the indicated temperatures. The numbers of animals scored are indicated in brackets. Statistical significance was determined with a Kruskal-Wallis non-parametric test followed by Dunn's multiple comparison correction and is indicated with ns for $p>0.05$ and **** for $p < 2.9e-7$. (**D**) Time-lapse imaging sequence of AC proliferation in a *zhIs150[pbx-ACELp>lin-39::gfp]* animal from the mid-L2 stage (24 hours post L1 starvation arrest) until young adulthood. Images are shown at the indicated time after start of the imaging experiment, with DIC images of mid-sagittal sections on top and maximum intensity projections of the LIN-39::GFP signal below. Yellow arrowheads indicate AC nuclei. See **Movie S4** for all time-points. The scale bar is 20 μm.

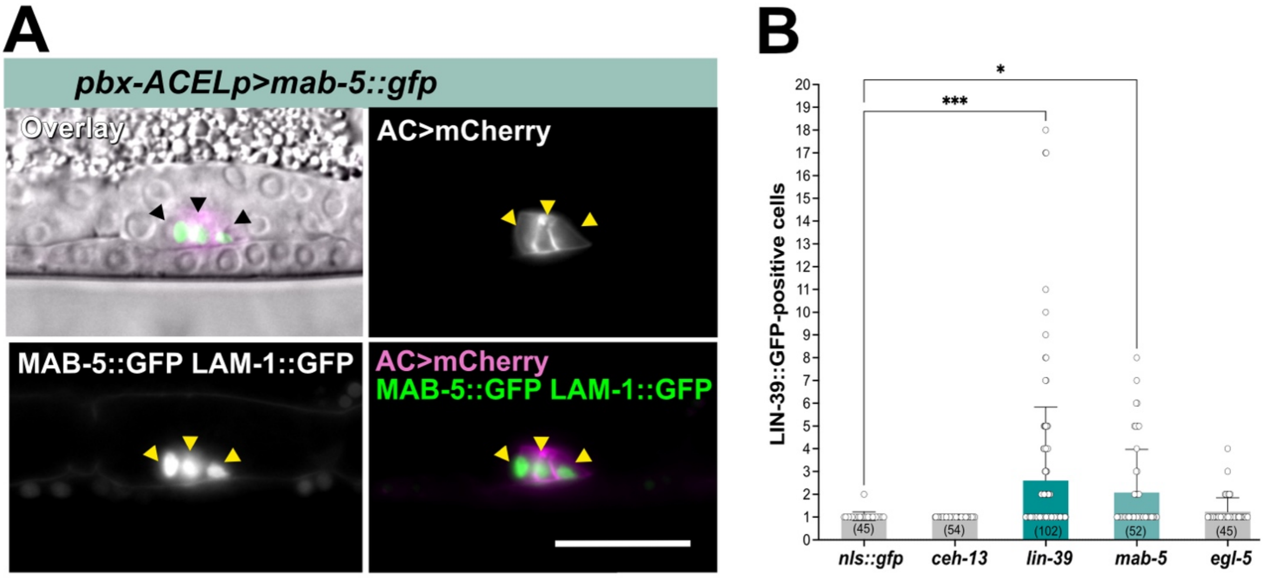

Figure S6 related to Figure 4.

(**A**) AC proliferation in a *zhEx660[pbx-ACELp>mab-5::gfp]* L3 larva. Mid-sagittal DIC image overlaid with the LAM-1::GFP (*qyIs10*) and MAB-5::GFP (green) and AC>mCherry (magenta) signals are shown, along with the separate and merged fluorescence channels. (**B**) AC number in L3 to L4 larvae for the indicated *hox* and *nls::gfp* transgenes. Bars show the average numbers of GFP-positive cells per animal, error bars the standard deviation and dots individual values. Two independent extra-chromosomal lines were analyzed for each transgene (*pbx-ACELp>nls::gfp*: *zhEx666.1 & 666.2*, *pbx-ACELp>ceh-13::gfp*: *zhEx662.1 & 662.2*, *pbx-ACELp>lin-39::gfp*: *zhEx650.1 & 650.2*, *pbx-ACELp>mab-5::gfp*: *zhEx660.1 & 660.2*, *pbx-ACELp>egl-5::gfp: zhEx661.1 & 661.2)*. AC nuclei are labeled with black (DIC) and yellow arrowheads. The numbers of animals scored are indicated in brackets. Statistical significance was determined with a Kruskal-Wallis non-parametric test followed by Dunn's multiple comparison correction and is indicated with * for p =0,017 and *** for p =4e-15. The scale bar is 20 µm.

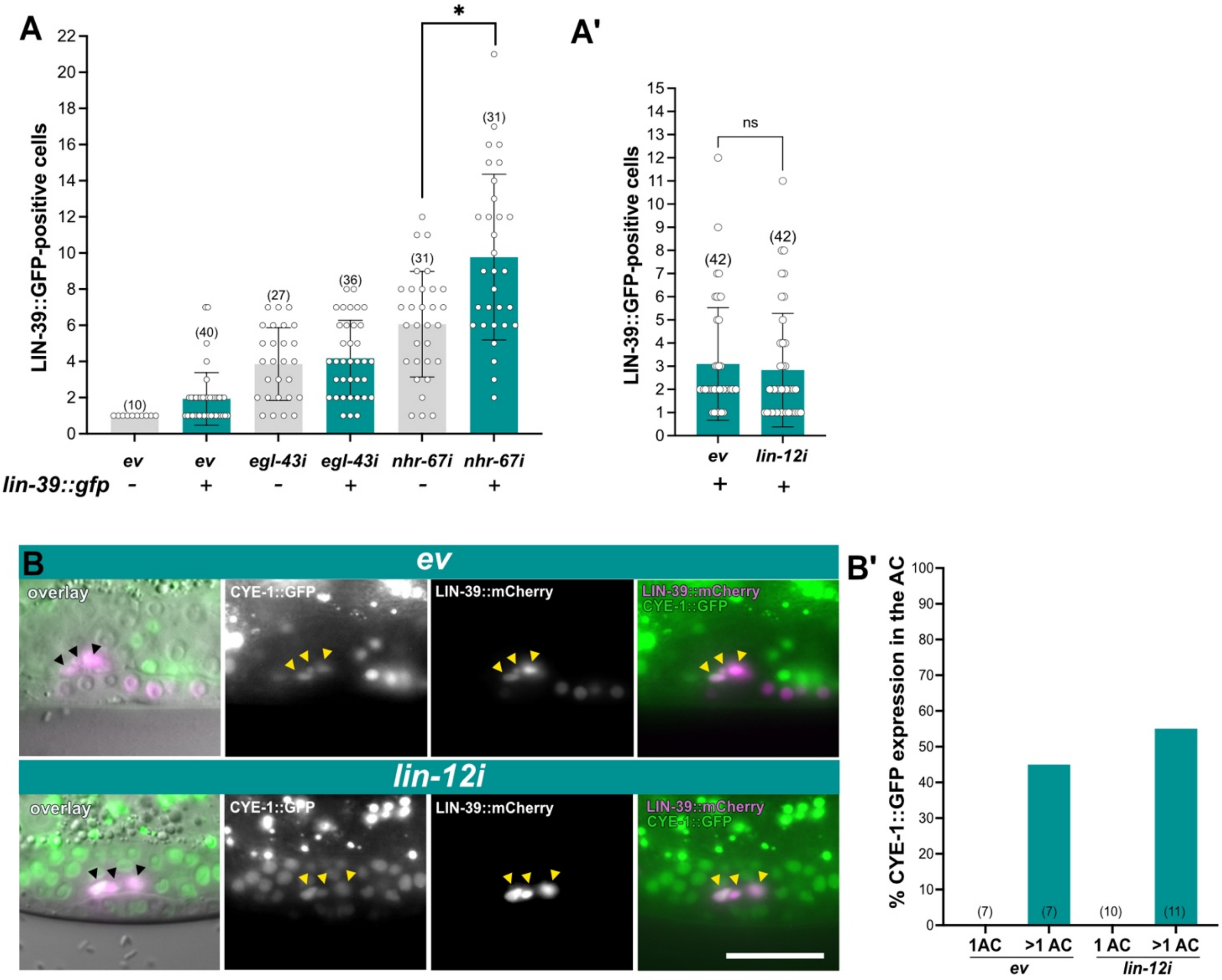

Figure S7 related to Figure 4.

(**A**) Quantification of AC proliferation in *zhIs150[pbx-ACELp>lin-39::gfp]* (+) and AC>mCherry (*qyIs23*) control (-) L3 larvae after *nhr-67*, *egl-43* or (**A'**) *lin-12* RNAi. (**B**) CYE-1::GFP (*zhIs80*) expression in *zhEx689[pbx-ACELp>lin-39::mCherry]* L3 larvae subjected to *lin-12i* and in ev controls. (**B'**) Quantification of CYE-1::GFP expression in *zhEx689[pbx-ACELp>lin-39::mCherry]* after control (ev) or *lin-12* RNAi in animals carrying a single or multiple ACs. Two independent *pbx-ACELp>lin-39::mCherry* lines (*zhEx689.1* & *689.2*) were analyzed. Arrowheads in (**B**) indicate AC nuclei. Black arrowheads in DIC images and white arrowheads in the fluorescent images indicate single and yellow arrowheads multiple ACs. Bar plots represent the average number of ACs in each population, error bars indicate the standard deviation and dots the individual values. Numbers of animals scored are indicated in brackets. Statistical significance was determined with a Mann-Whitney non-parametric test and is indicated with ns for p>0.05 and * for p =0.013. The scale bar is 20 μm.

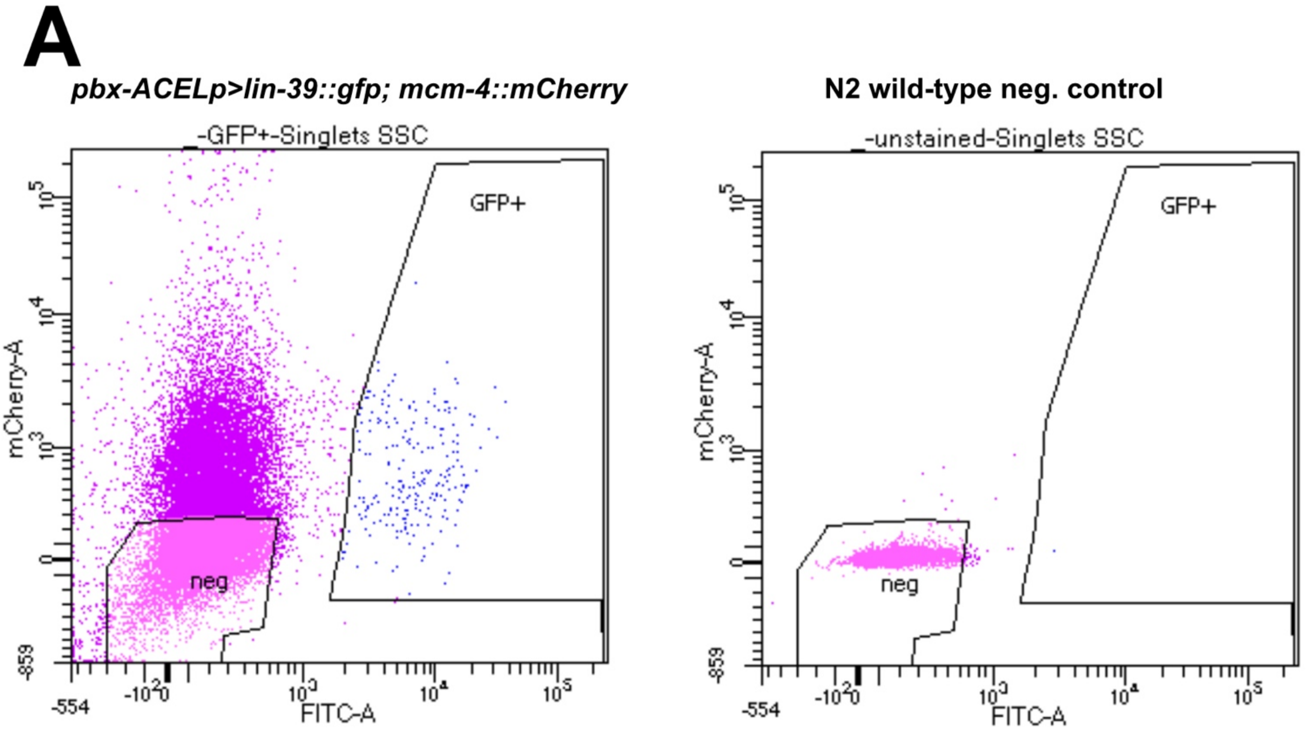

Tube: GFP+

Population	#Events	%Parent	%Total
All Events	110,000	####	100.0
cells	43,413	39.5	39.5
singlets FSC	36,181	83.3	32.9
Singlets SSC	30,830	85.2	28.0
GFP+	236	0.8	0.2
neg	16,920	54.9	15.4

Tube: unstained

Population	#Events	%Parent	%Total
All Events	10,000	####	100.0
cells	4,379	43.8	43.8
singlets FSC	3,873	88.4	38.7
Singlets SSC	3,683	95.1	36.8
GFP+	1	0.0	0.0
neg	3,642	98.9	36.4

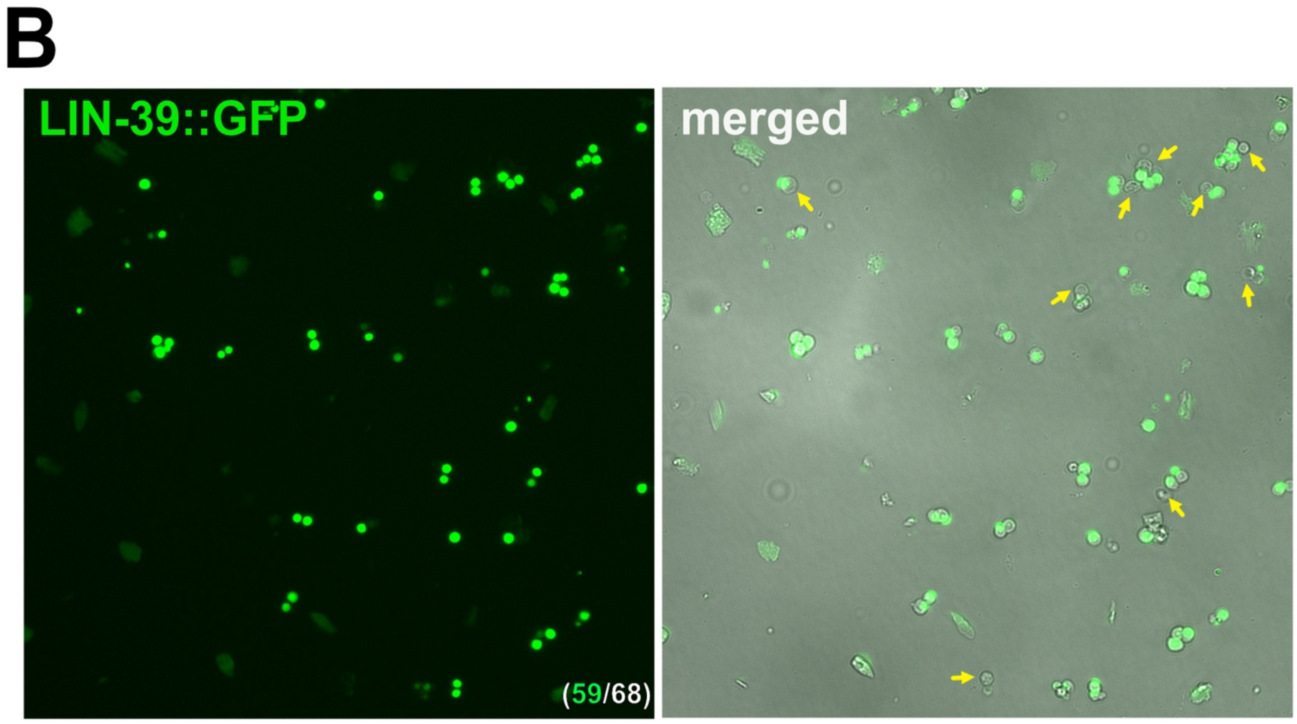

Figure S8 related to Figure 4.

(**A**) FACS sorting of cells isolated from *zhIs150[pbx-ACELp>lin-39::gfp]; heSi45[mcm-4::mCherry]* (left) and N2 larvae used as negative controls (right). The analysis of cells used for Expt3 (**Tab. S1**) are shown. A fraction of the cells was first analyzed to select LIN-39::GFP-positive cells, and the remaining cells were directly collected in RNA extraction buffer. In each experiment, LIN-39::GFP & MCM-4::mCherry-negative cells were collected as non-proliferating control population. For the analysis of all three experiments and the raw FACS data, see the Source Data file. (**B**) Example of LIN-39::GFP-positive FACS-sorted cells in an independent experiment that were plated in L15 medium supplemented with 10% bovine fetal calf serum on collagen-coated glass slides. The right panel shows the phase-contrast image overlaid with the LIN-39::GFP signal. Yellow arrows point to LIN-39::GFP-negative cells. In this example, approximately 59 out of 68 cells expressed LIN-39::GFP.

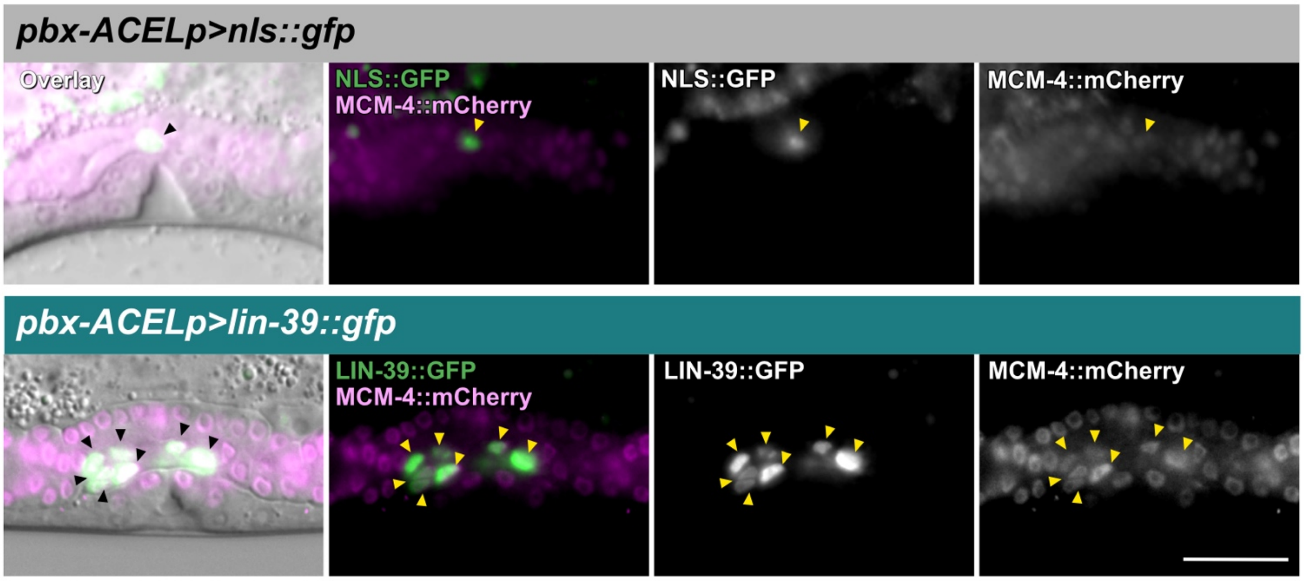

Figure S9 related to Figure 5.

MCM-4::mCherry expression in *pbx-ACEL>nls::gfp* (top panels) and *pbx-ACEL>lin-39::gfp* L4.1 larvae (before AC fusion) [32]. Mid-sagittal DIC image overlaid with the NLS::GFP or LIN-39::GFP (green) and MCM-4::mCherry (magenta) signals are shown, along with the separate and merged fluorescence channels. The yellow arrowheads indicate nuclei co-expressing LIN-39::GFP and MCM-4::mCherry. See **Fig. 5C**' for the numbers of animals observed for each condition. The scale bar is 20μm.
