# Supplementary material for: Prolonging somatic cell proliferation through constitutive hox gene expression in C. elegans
Source: Nat Commun. 2023 Oct 27;14:6850. doi: 10.1038/s41467-023-42644-1 (PMC10611754; doi:10.1038/s41467-023-42644-1)
Supplement: Supplementary file 3 — Description of Additional Supplementary Files [file 41467_2023_42644_MOESM3_ESM.pdf]

Description of Additional Supplementary Files

Supplementary Data Legends

Supplementary Data S1. Results of the AC transcriptome analysis. The raw, two-tailed p-values and log2Ratios were calculated from the three repeats of each condition (GFP-positive vs GFP-negative) using the DESeqResults function.

Supplementary Data S2. List of *C. elegans* strains used.

Supplementary Data S3. List of oligonucleotides used.

Supplementary Data S4. List of plasmids used.

Supplementary Movie legends

Supplementary Movie S1. Time-lapse imaging sequence of LIN-39::GFP expression during larval development.

Supplementary Movie S2. Time-lapse imaging sequence of the *zhIs167[egl-17p>lin-39::gfp]* larva with early Pn.p cell duplications but normal vulval induction shown in Fig. S3A.

Supplementary Movie S3. Time-lapse imaging sequence of the *zhIs167[egl-17p>lin-39::gfp]* larva with early Pn.p cell duplications and over-induction shown in Fig. S3B.

Supplementary Movie S4. Time-lapse imaging sequence of AC proliferation in the *zhIs150[pbx-ACELp>lin-39::gfp]* animal shown in Fig. S5D.

Supplementary Movie S5. Time-lapse imaging sequence of AC proliferation in the *zhIs150[pbx-ACELp>lin-39::gfp]* animal shown in Fig. 5A.

Supplementary Movie S6. Time-lapse imaging sequence of AC proliferation in the *aff-1(lf); fem-2(ts); zhIs150[pbx-ACELp>lin-39::gfp]* animal shown in Fig. 5B.
