# Supplementary material for: Prolonging somatic cell proliferation through constitutive hox gene expression in C. elegans
Source: Nat Commun. 2023 Oct 27;14:6850. doi: 10.1038/s41467-023-42644-1 (PMC10611754; doi:10.1038/s41467-023-42644-1)
Supplement: Supplementary file 15 — Source data [file 41467_2023_42644_MOESM15_ESM.zip › FACS data/EXPT1/EXPT1_analysis.pdf]

# BD FACSDiva 8.0.1

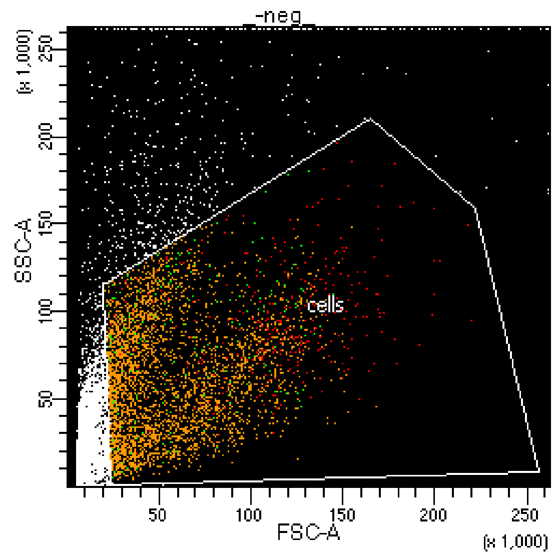


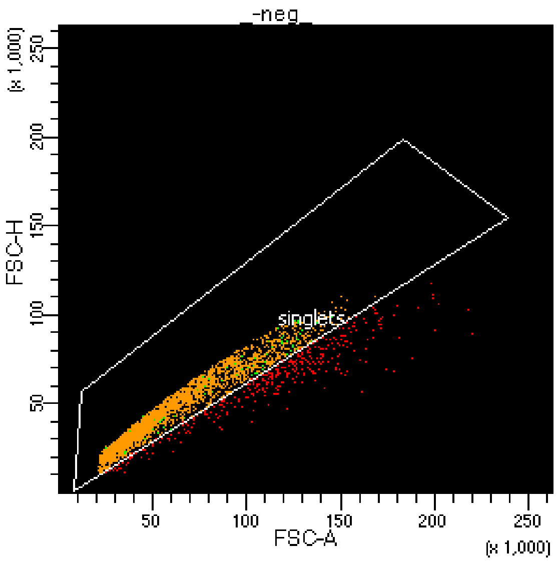


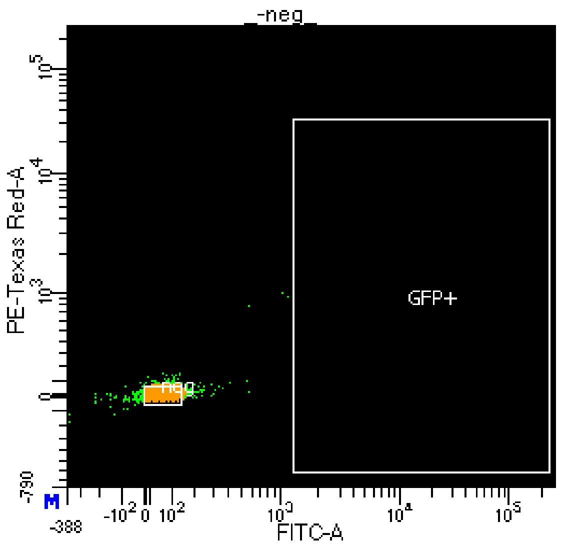


Tube: neg_

| Population | #Events | %Parent | %Total |
|---|---|---|---|
| All Events | 10,000 | #### | 100.0 |
| cells | 3,585 | 35.8 | 35.8 |
| singlets | 3,199 | 89.2 | 32.0 |
| GFP+ | 0 | 0.0 | 0.0 |
| neg | 2,941 | 91.9 | 29.4 |

# BD FACSDiva 8.0.1

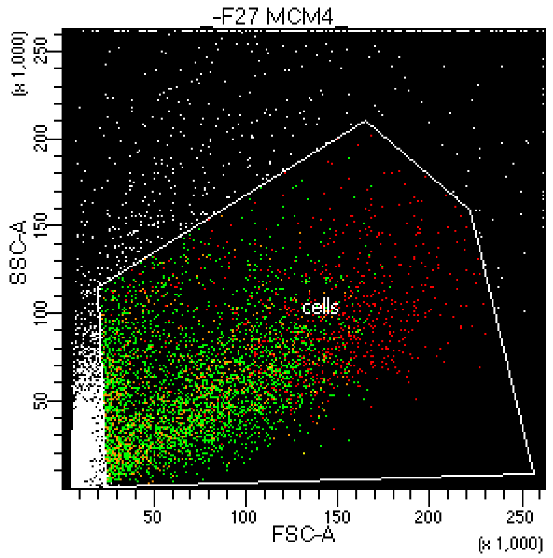


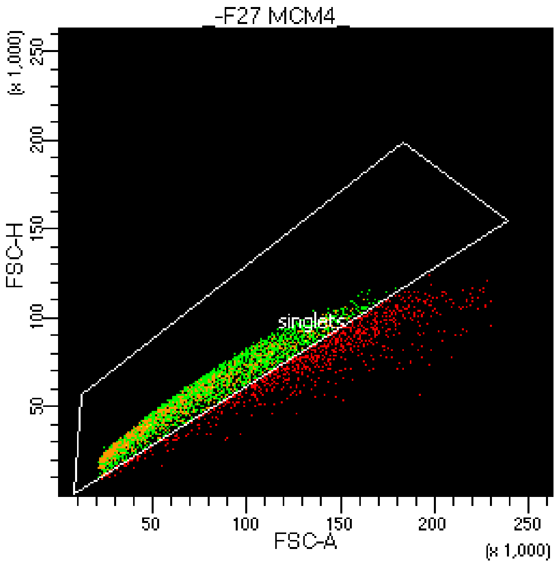


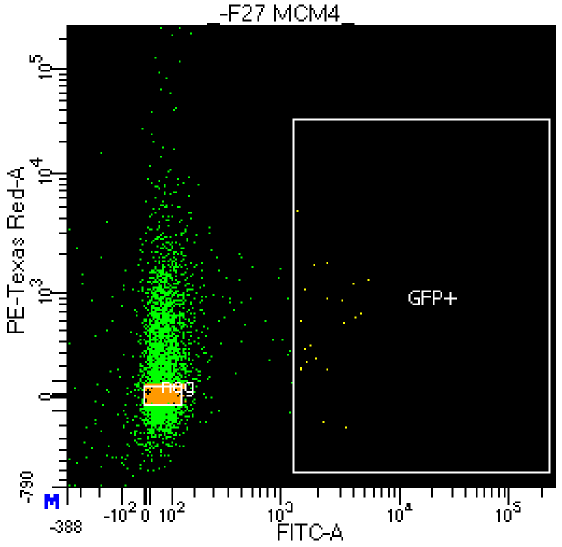


Tube: F27 MCM4_

| Population | #Events | %Parent | %Total |
|---|---|---|---|
| All Events | 10,000 | #### | 100.0 |
| cells | 4,406 | 44.1 | 44.1 |
| singlets | 3,592 | 81.5 | 35.9 |
| GFP+ | 21 | 0.6 | 0.2 |
| neg | 712 | 19.8 | 7.1 |
