# Supplementary material for: Prolonging somatic cell proliferation through constitutive hox gene expression in C. elegans
Source: Nat Commun. 2023 Oct 27;14:6850. doi: 10.1038/s41467-023-42644-1 (PMC10611754; doi:10.1038/s41467-023-42644-1)
Supplement: Supplementary file 15 — Source data [file 41467_2023_42644_MOESM15_ESM.zip › FACS data/EXPT3/EXPT3_analysis.pdf]

Batch Analysis Report

Run Date: 12/9/21 4:02 PM

Experiment: 2021-12-09 Michael Daube

User ID: DivaUser

Statistics Output: N/A

Worksheet PDF Output: \\tsclient\Z\fcfstaff\SorterService\m.daube\2021-12-09 Michael Daube-Batch_Analysis_09122021160209.pdf

_

Tube	Status	Run Time
unstained	OK	12/9/21 4:02 PM
GFP+	OK	12/9/21 4:02 PM

BD FACSDiva 8.0.1

_-unstained

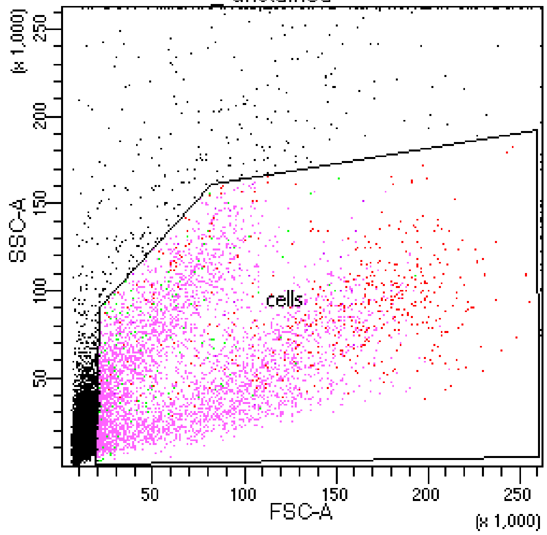

_-unstained-cells

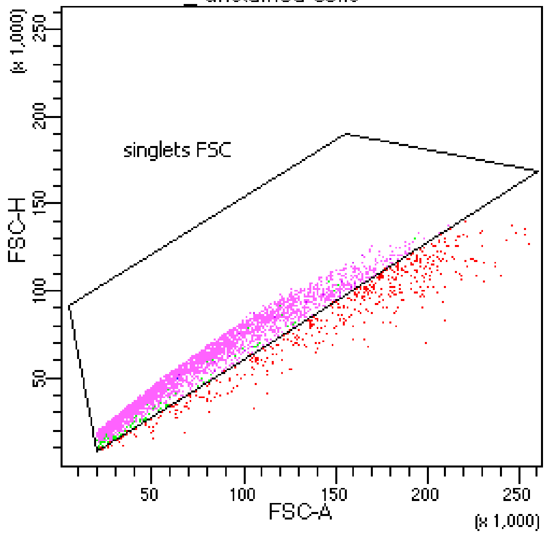

_-unstained-singlets FSC

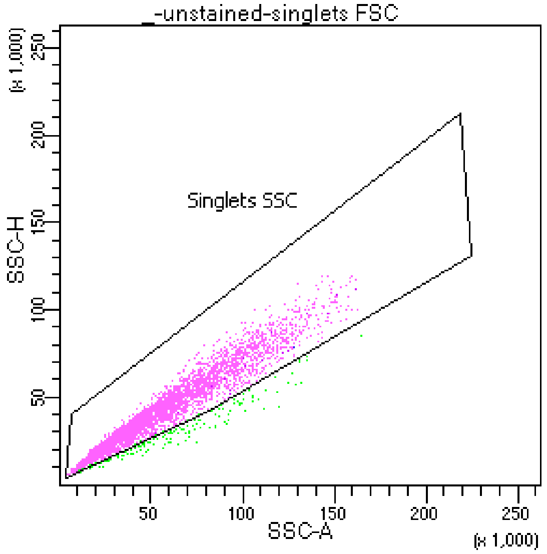

_-unstained-Singlets SSC

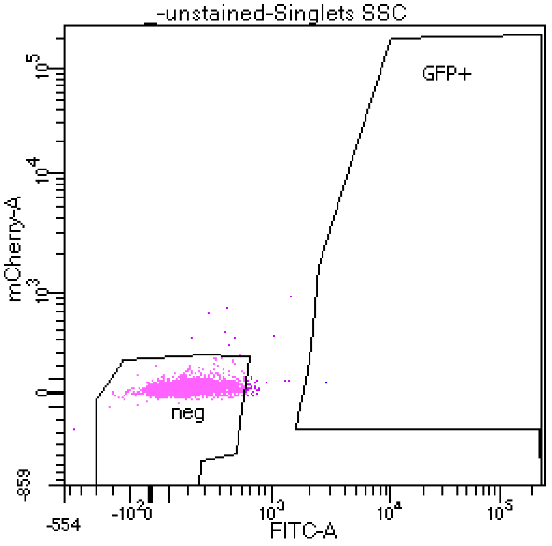

Tube: unstained

Population	#Events	%Parent	%Total
All Events	10,000	####	100.0
cells	4,379	43.8	43.8
singlets FSC	3,873	88.4	38.7
Singlets SSC	3,683	95.1	36.8
GFP+	1	0.0	0.0
neg	3,642	98.9	36.4

Printed on: Thu Dec 9, 2021 04:02:22 CET

BD FACSDiva 8.0.1

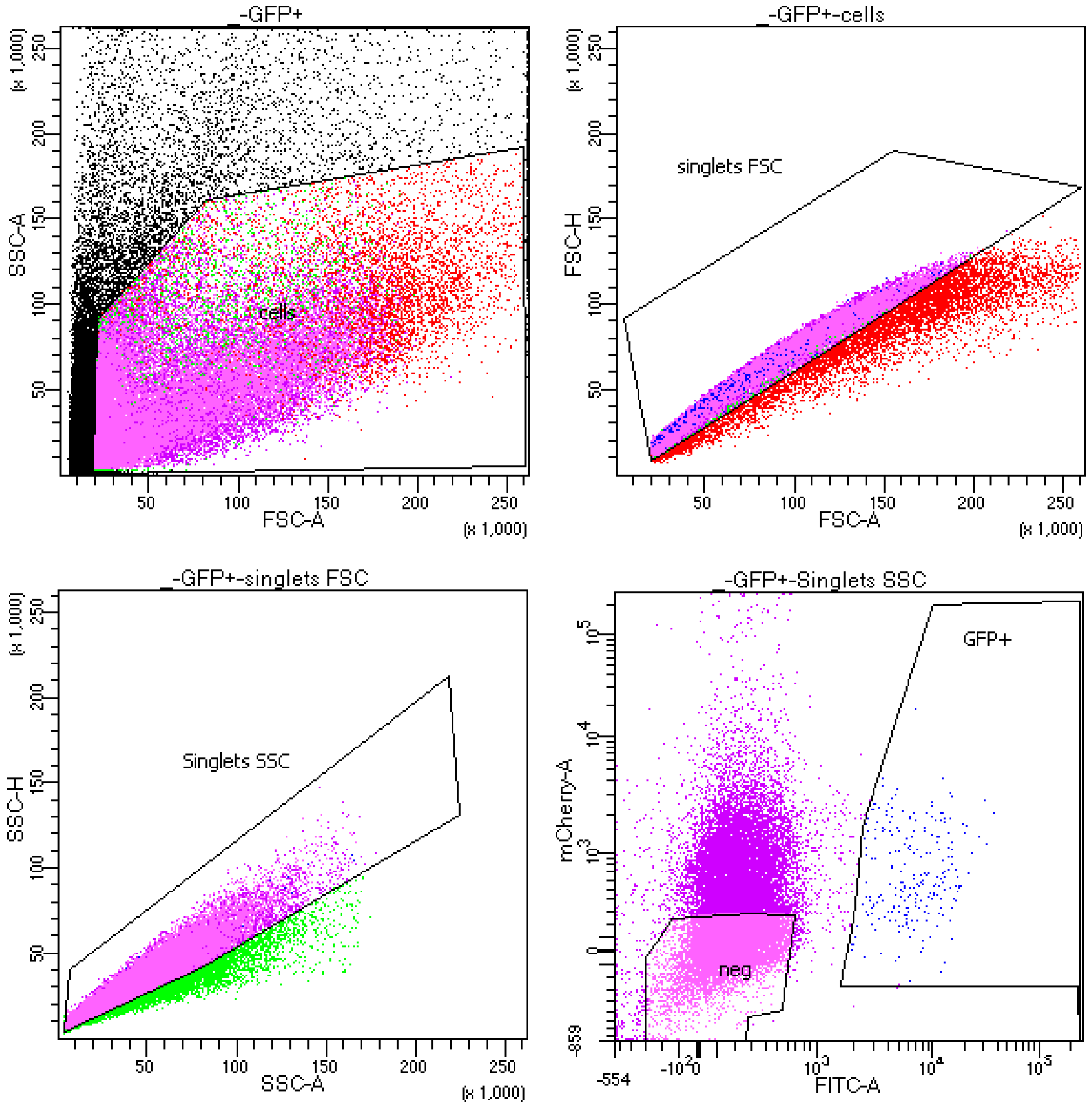

Tube: GFP+

Population	#Events	%Parent	%Total
All Events	110,000	####	100.0
cells	43,413	39.5	39.5
singlets FSC	36,181	83.3	32.9
Singlets SSC	30,830	85.2	28.0
GFP+	236	0.8	0.2
neg	16,920	54.9	15.4
