# Supplementary material for: Prolonging somatic cell proliferation through constitutive hox gene expression in C. elegans
Source: Nat Commun. 2023 Oct 27;14:6850. doi: 10.1038/s41467-023-42644-1 (PMC10611754; doi:10.1038/s41467-023-42644-1)
Supplement: Supplementary file 15 — Source data [file 41467_2023_42644_MOESM15_ESM.zip › FACS data/EXPT2/EXP2_analysis.pdf]

Batch Analysis Report

Run Date: 12/8/21 4:51 PM

Experiment: 2021-12-08 Michael Daube

User ID: DivaUser

Statistics Output: N/A

Worksheet PDF Output: \\tsclient\Z\fcfstaff\SorterService\m.daube\2021-12-08 Michael Daube-Batch_Analysis_08122021165107.pdf

_

Tube	Status	Run Time
unstained	OK	12/8/21 4:51 PM
GFP	OK	12/8/21 4:51 PM

BD FACSDiva 8.0.1

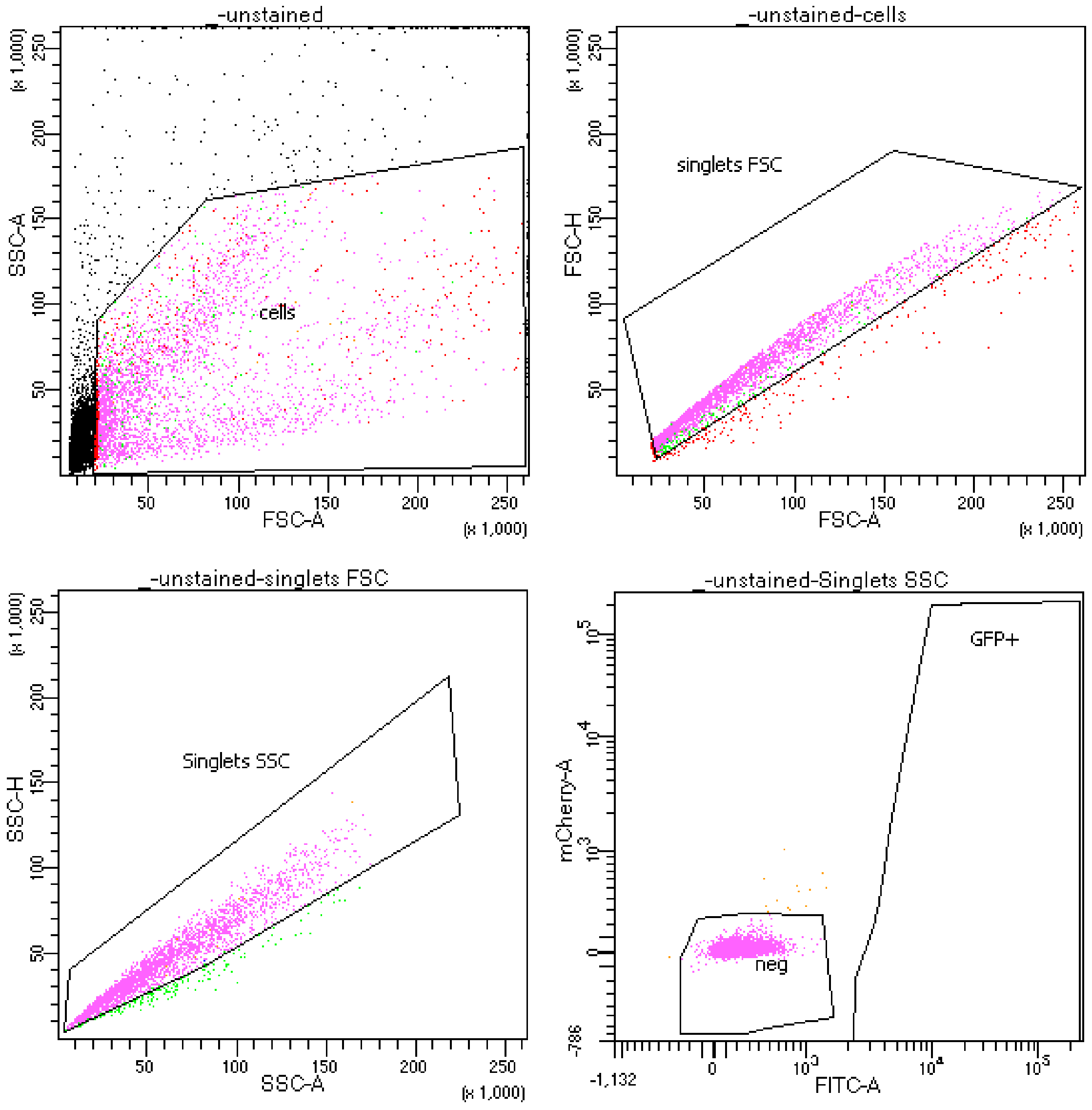

Tube: unstained

Population	#Events	%Parent	%Total
All Events	10,000	####	100.0
cells	3,535	35.4	35.4
singlets FSC	3,089	87.4	30.9
Singlets SSC	2,880	93.2	28.8
GFP+	1	0.0	0.0
neg	2,864	99.4	28.6

BD FACSDiva 8.0.1

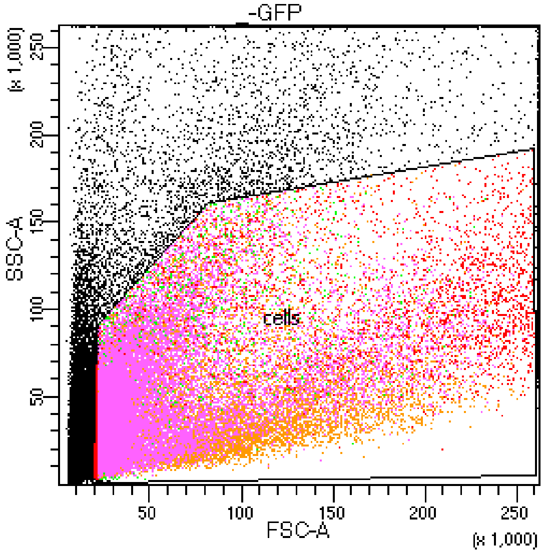

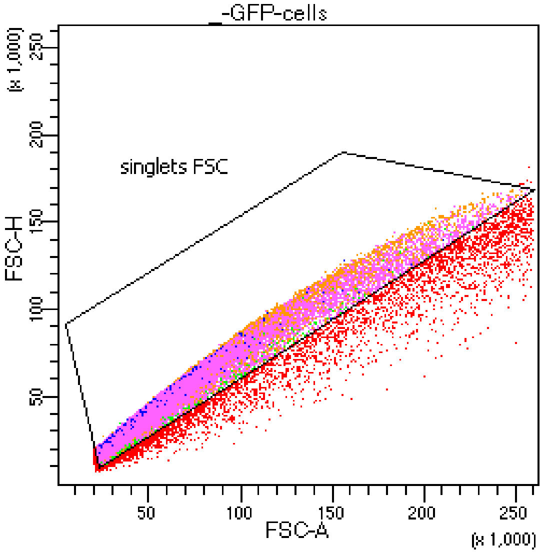

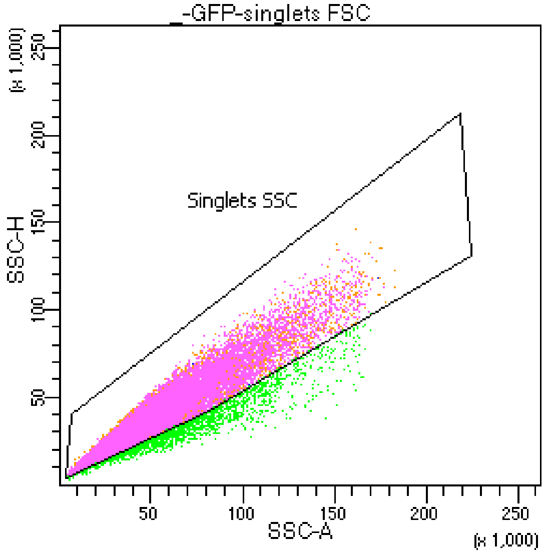

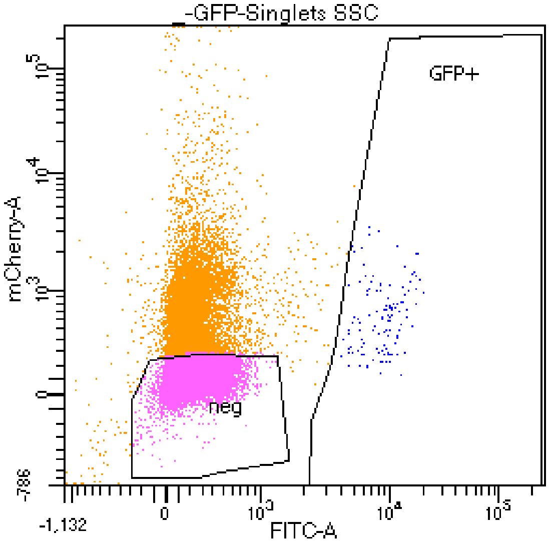

Tube: GFP

Population	#Events	%Parent	%Total
All Events	110,000	####	100.0
cells	33,808	30.7	30.7
singlets FSC	28,242	83.5	25.7
Singlets SSC	25,821	91.4	23.5
GFP+	121	0.5	0.1
neg	16,009	62.0	14.6
